# Use of antagonist muscle EMG in the assessment of neuromuscular health of the low back

**DOI:** 10.1186/s40101-015-0055-5

**Published:** 2015-04-24

**Authors:** Nakyung Lee, Hwayeong Kang, Gwanseob Shin

**Affiliations:** Department of Human and Systems Engineering, Ulsan National Institute of Science and Technology, Ulju-gun, Ulsan 689-798 Korea

**Keywords:** Surface electromyography, EMG, Low back pain, Lumbar spine, Biomechanics, Spine stability

## Abstract

**Background:**

Non-specific low back pain (LBP) has been one of the most frequently occurring musculoskeletal problems. Impairment in the mechanical stability of the lumbar spine has been known to lower the safety margin of the spine musculature and can result in the occurrence of pain symptoms of the low back area. Previously, changes in spinal stability have been identified by investigating recruitment patterns of low back and abdominal muscles in laboratory experiments with controlled postures and physical activities that were hard to conduct in daily life. The main objective of this study was to explore the possibility of developing a reliable spine stability assessment method using surface electromyography (EMG) of the low back and abdominal muscles in common physical activities.

**Methods:**

Twenty asymptomatic young participants conducted normal walking, plank, and isometric back extension activities prior to and immediately after maintaining a 10-min static upper body deep flexion on a flat bed. EMG data of the erector spinae, external oblique, and rectus abdominals were collected bilaterally, and their mean normalized amplitude values were compared between before and after the static deep flexion. Changes in the amplitude and co-contraction ratio values were evaluated to understand how muscle recruitment patterns have changed after the static deep flexion.

**Results:**

Mean normalized amplitude of antagonist muscles (erector spinae muscles while conducting plank; external oblique and rectus abdominal muscles while conducting isometric back extension) decreased significantly (*P* < 0.05) after the 10-min static deep flexion. Normalized amplitude of agonist muscles did not vary significantly after deep flexion.

**Conclusions:**

Results of this study suggest the possibility of using surface EMG in the evaluation of spinal stability and low back health status in simple exercise postures that can be done in non-laboratory settings. Specifically, amplitude of antagonist muscles was found to be more sensitive than agonist muscles in identifying changes in the spinal stability associated with the 10-min static deep flexion. Further research with various loading conditions and physical activities need to be performed to improve the reliability and utility of the findings of the current study.

## Background

Low back pain (LBP) has been one of the most frequently occurring musculoskeletal disorders, with the lifetime prevalence of as high as 84% [1]. While LBP can result from various risk factors, majority of LBP cases do not have clear causes and often be categorized as non-specific [2]. Although it is difficult to identify causal factors and injury mechanisms of the non-specific LBP, it is commonly accepted that the impairment in the mechanical stability of the lumbar spine musculature can influence muscle recruitment patterns and contribute to the occurrence of the non-specific LBP symptoms [3].

Mechanical stability of the lumbar spine is often interpreted as the level of tolerance or safety margin that the lumbar spine can resist against external perturbations [4,5]. The spinal stability is known to be maintained by coordinated contributions of passive tension forces from passive spinal tissues as well as active contraction forces from the low back and abdominal muscles around the lumbar spine [3,6]. Risk factors that are known to damage the stability include acute inflammation of spinal ligaments [7], reduced stiffness of passive tissues due to prolonged or repetitive stooped posture [8,9], and fatigue development of the low back muscles [10].

Minor changes in spinal stability may not directly lead to pain symptoms, but it can reduce the safety margin of the lumbar spine and make the lumbar spine more vulnerable to injuries or disorders. To prevent the occurrence of non-specific LBPs due to damages in the spinal stability, reliable assessment of the level of spinal stability prior to the occurrence of pain symptoms becomes essential.

In previous research, lumbar spine stability has been evaluated by identifying abnormal patterns of myoelectric (EMG) signals of the low back muscles or computing spinal stiffness by numerical modeling with trunk kinematics analysis. Typical EMG indicators of the changes in the level of spinal stability include the delayed occurrence of the flexion-relaxation phenomenon of the low back extensor muscles in trunk flexion [11], increase in the co-contraction ratio between the abdominal and low back muscles [12,13], increase in the frequency and strength of muscle spasms [7], and the amount of bilateral imbalance of the low back muscle activity in weight holding [14].

Although these indicators have shown good validity in determining the asymptomatic damages in spinal stability, they have been typically evaluated in controlled experiments with restrained movements and guided postures that might not be easily repeated in non-laboratory environments [15,16]. Use of these indicators in non-laboratory environments is still not practical due to the lack of standardized methods or difficulties in conducting controlled movements and postures. If the indicators of spinal stability can be reliably collected and evaluated in non-laboratory settings with simple and easy-to-perform tasks, it could lead to the development of a spine health assessment system for point-of-care by clinicians or self-care by individuals.

As the first step for the development of a spine health assessment model (data collection protocol and analysis algorithm) for clinicians and self-care of individuals, the current study was aimed specifically to determine specific stability indicators that are sensitive in differentiating damaged lumbar spine from healthy lumbar spine, and standardized data collection protocols that can be safely and reliably conducted in non-laboratory environments. Potential applications of this research include the development of wearable spine health monitoring systems that can identify the changes in injury tolerance of the low back and warn the wearer of the high risk of acute low back injuries.

## Methods

### Data collection

Twenty young individuals (10 females, 10 males) between 19 and 35 years old who had no history of low back disorders and current pain symptoms on the low back were recruited from the university population (Table 1). Individuals who were incapable of conducting typical physical exercises and who could not maintain deep flexion posture were excluded. All participants provided written consent on a protocol approved by the institutional review board of the Ulsan National Institute of Science and Technology (UNIST) prior to participation.

**Table 1:** **Subject information (mean and standard deviation)**

	**Age (years)**	**Height (cm)**	**Weight (kg)**
Female (*n* = 10)	19.8 (0.92)	161.9 (5.2)	55.0 (4.0)
Male (*n* = 10)	20.0 (2.00)	175.5 (3.6)	65.6 (5.6)
All	19.9 (1.52)	168.7 (8.2)	60.3 (7.2)

Each participant performed a set of physical exercises before and immediately after maintaining a deep upper body flexion posture for 10 min on a flat bed. EMG signals of the low back and abdominal muscles were collected during the exercises and then compared between before and after the deep flexion task to determine whether changes in muscle activation patterns associated with the prolonged deep flexion could be identified during the physical exercises.

**Figure 1:**
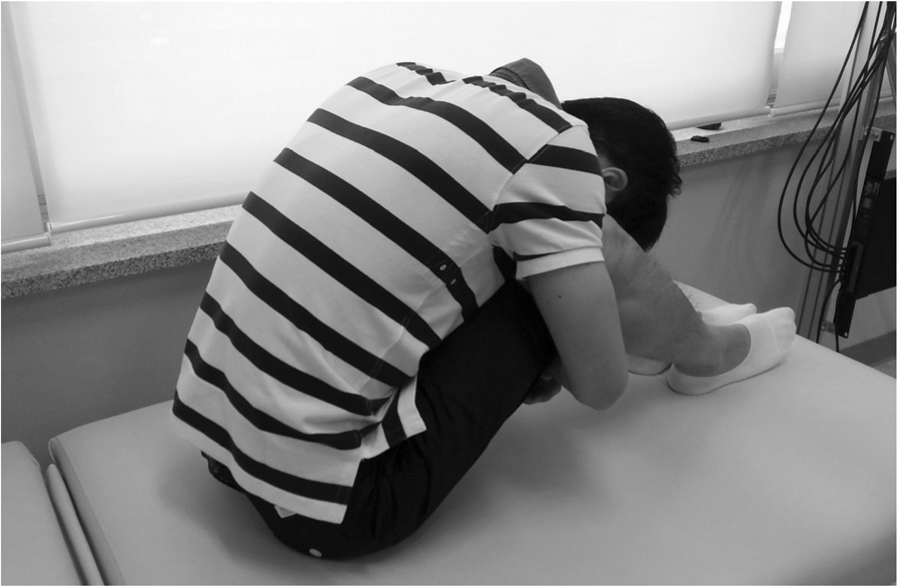
Static deep flexion posture.

During the deep flexion task, the participant fully flexed the upper body on a cushioned table and maintained the posture for 10 min continuously. Knee flexion was allowed to relieve the tension on the hamstrings (Figure 1). In previous research, it has been shown that maintaining the deep flexion posture for 10 min could briefly increase the laxity in the spine musculature and result in acute damage in the spinal stability [16]. In the current study, the same 10-min deep flexion protocol was employed to incur mild and pain-free status of spinal instability.

**Figure 2:**
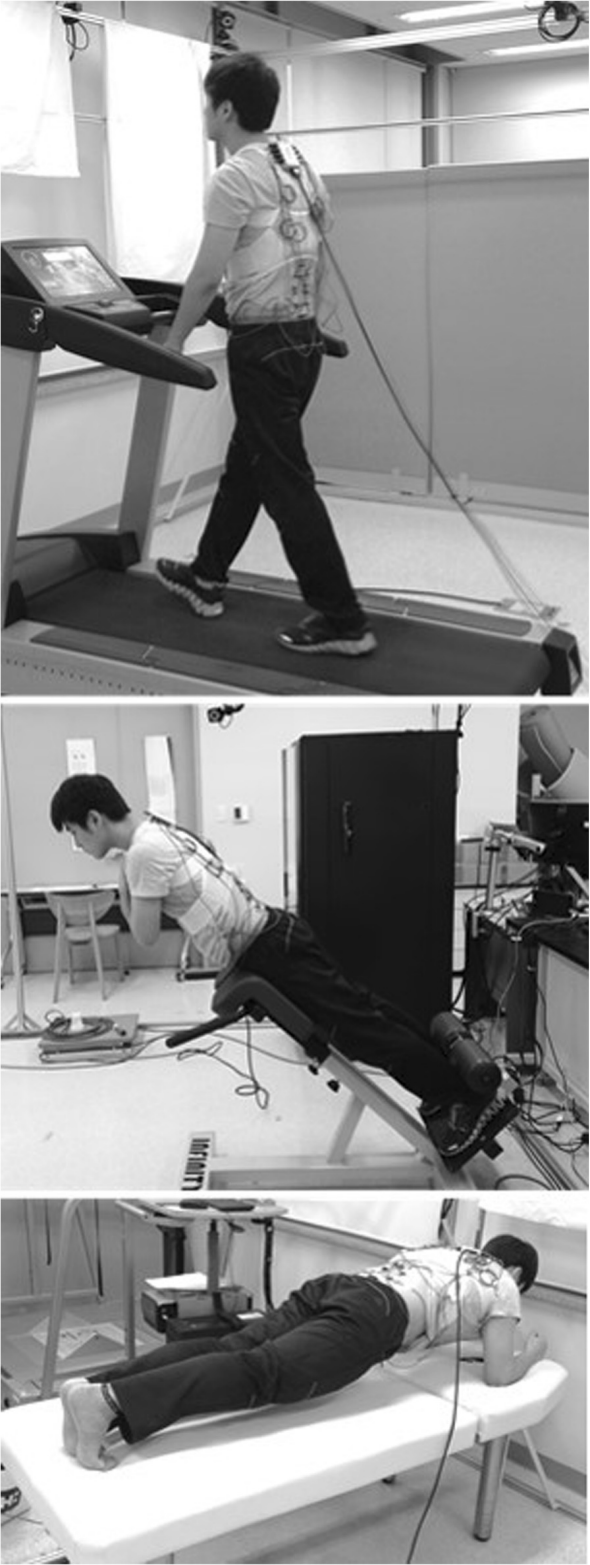
Walking (top), isometric back extension (middle), and plank (bottom) exercises.

Physical exercises that were conducted immediately before and after the 10-min deep flexion included ‘walking at a normal speed on a treadmill’, ‘maintaining the plank posture (prone bridge)’, and ‘maintaining an isometric back extension posture on a roman chair’. For the walking exercise, the participant walked for 60 s on a treadmill at his/her normal speed (4 ~ 6 km/h) with both arms swinging naturally. During the plank and isometric back extension exercises, the participant was asked to maintain proper postures for 10 s as instructed by the experimenter and keep breathing normally (Figure 2). The three exercises were selected specifically as they are common physical exercises that can be done easily in non-laboratory environments and they require coordinated activation of both trunk extensors and flexors. EMG data were recorded for the middle 10 s while walking and the middle 5 s while maintaining the plank and isometric back extension postures.

EMG data were collected from eight surface electrodes around the lumbar spine. Electrodes were attached bilaterally to the erector spinae muscles (ES, L2, and L4 levels), external oblique (EO) muscles, and rectus abdominals (RA) using double-sided adhesive tapes (Table 2). The specific locations of the electrodes were determined, based on findings of previous research, to collect EMG data from co-contracting muscles during the tested physical exercises [8].

**Table 2:** **Electrode locations**

**Muscle**	**Electrode location**
Erector spinae	3.5 cm from the midline at the level of L2 and L4 vertebrae
External oblique	Approximately 10 cm lateral to umbilicus with an orientation of 45° from vertical
Rectus abdominals	3 cm lateral to the umbilicus

Raw EMG signals were collected at 2,048 Hz using single differential bipolar electrodes (Bagnoli 2.1, Delsys, MA, USA) with an input impedance of greater than 10^15^ Ω, a channel bandwidth of 20 to 450 Hz, and a common mode rejection ratio of 84 dB. Inter-electrode distance was 1 cm. Signals were band-pass filtered, full-wave rectified, and then smoothed using a second-order Butterworth filter with a low-pass cut-off frequency of 3 Hz to produce the linear envelope EMG.

To generate normalized EMG amplitude values, the smoothed EMG data of the erector spinae muscles of each exercise were then divided by the mean EMG amplitude of the same muscle of the initial isometric back extension exercise that was performed before the 10-min deep flexion. EMG data of the abdominal muscles were normalized by the mean amplitude of the same muscle from the initial plank exercise. The sub-maximum EMG amplitudes were chosen as the nominators for EMG normalization because maximum voluntary contraction protocols often require trained experimenters and dedicated equipment to warrant participant’s safety and data reliability, which are difficult in non-laboratory environments. The normalized EMG (NEMG) data of each channel were then averaged over the recording duration of each exercise.

### Data analysis

Since no significant bilateral difference was found from each pair of muscle, NEMG data of each muscle pair were pooled for subsequent analyses. The mean NEMG values were then compared between pre- and post-deep flexion exercises by one-way analysis of variance (ANOVA) with repeated measures to determine whether the 10-min static upper body deep flexion caused significant changes in the dependent variables. A significance criterion of *P* < 0.05 was used for all statistical analyses.

## Results

**Figure 3:**
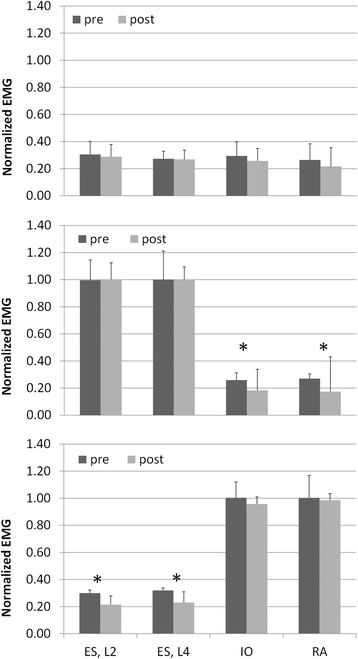
Mean normalized EMG before and after static deep flexion. Walking (top), isometric back extension (middle), and plank (bottom) exercises. Bars above mean columns indicate 1-standard deviation. *P < 0.05. EMG, electromyography.

Mean NEMG of the lumbar erector spinae muscles and the abdominal muscles decreased after the 10-min deep flexion in general, but statistically significant (*P* < 0.05) decrements were found only from antagonist muscles in the plank and isometric back extension exercises (Figure 3). That is, the erector spinae muscles produced significantly lower NEMG after deep flexion while conducting the plank exercise. The external oblique and rectus abdominal muscles generated significantly lower NEMG after deep flexion while conducting the isometric back extension exercise. No significant effect of deep flexion was found from the walking activity for all muscles.

## Discussion

In this study, it was found that the amount of muscle activation of the lumbar erector spinae muscles and the abdominal muscles while performing isometric contraction activities could vary after 10-min static deep flexion, and the difference was more pronounced for the antagonist muscles in plank and isometric back extension exercises.

The 10-min static upper body deep flexion task was included in this study as a physically challenging activity that was believed to cause joint laxity and slightly damage spinal stability without pain symptoms. In previous research, prolonged stooping or deep flexion has been found to affect the stress–strain relationship of spinal tissues such as posterior ligaments and passive components of muscle-tendon units [11,17]. Static elongation of the posterior spinal tissues in deep flexion is known to decrease the stiffness of posterior spinal ligaments, and it can cause the low back extensor muscles to generate greater contraction forces to compensate for the reduced tissue stiffness of posterior ligaments [11].

In the current study, however, the level of activity of the lumbar erector spinae muscles did not increase after deep flexion, and it might be attributable to the straight back posture in the isometric back extension. Different from previous research where participants conducted lifting or weight-holding tasks in forward flexed postures [18], the participants of the current study made a straight back posture on a roman chair. Since posterior ligaments were less stretched in the stretched back posture compared to when maintaining flexed postures, most of lumbar extension moments during the isometric back extension exercise might have been produced by the active contraction of extensor muscles, minimizing the influence of the reduced stiffness of posterior spinal ligaments on the activation level of the low back extensors.

Significant effects of the 10-min static deep flexion were found on the activation level of antagonist muscles in the isometric back extension. The rectus abdominals and the external oblique muscles were antagonist muscles in the isometric back extension as their activation generated a flexion moment around the lumbar spine while the low back extensors were maintaining the posture by producing an extension moment. Synchronous activation of the agonist and antagonist muscles around a joint helps individuals control movements and/or maintain postures and is known to improve the stability of the joint [12]. The reduced co-activation of the abdominal muscles after the static deep flexion could be indicative of the changes in spinal stability.

Decrease in the antagonist muscle activation level was also observed in the plank as well. The straight upper body of the plank exercise is maintained primarily by the upper body flexion moment from the contraction forces of abdominal muscles. In literature, it has been reported that the lumbar erector spinae muscles and other low back extensor muscles produce contraction forces of less than 10% of their maximum voluntary contraction capacity and contribute to the spinal stability when conducting the plank exercise [19,20]. Similar to the abdominal muscles in the isometric back extension, the decrease in the activation level of the lumbar erector spinae muscles in the plank might be attributable to the 10-min deep flexion and resultant changes in spinal stability.

The co-activation of antagonist muscles is known as the involuntary activation during the voluntary contraction of agonist muscles. The role of antagonist co-activation in maintaining joint stability has been consistently addressed in previous research, and it has been known that the joint stiffness or stability could be improved with greater co-activation of antagonist muscles [21-23]. It has also been found that individuals with damaged joint stability due to muscle fatigue would generate greater co-activation of antagonist muscles to compensate for the reduced stability of the joint [24].

The results of the current study, however, did not comply with the findings in previous research, and it might be attributable to the difference in the main cause of stability impairment. Different from previous research where damage in the muscle contraction performance caused the instability of the lumbar spine, the current study tested the effects of passive tissue stretching and resultant increase in the joint laxity. The prolonged elongation of posterior ligaments during the 10-min deep flexion might have affected the motor control of adjacent core muscles of the lumbar spine, resulting in the inefficiency of antagonist co-activation [25]. However, it should be noted that additional explanations need further research, specifically on the control mechanism of co-activation of antagonist muscles.

Analysis and interpretation of surface EMG signals in kinesiological EMG and ergonomics areas have been focused mainly on agonist muscles that generate major moments around the joint of interest. Synchronous activation of antagonist muscles has not been considered frequently due to the relatively weak activation level of the antagonist muscles. The results of this study suggest that the responses of antagonist muscles, which have not received much attention before, could be more sensitive than agonist muscles in identifying minor changes in spinal stability.

Compared to previous research [11,17], the 10-min static deep flexion of this study was relatively mild in terms of its physical intensity. It might have caused smaller changes in the spinal stability compared to what have been observed in previous research that tested more physically challenging postures and activities. While major agonist muscles such as the lumbar erector spinae muscles in the isometric back extension and the rectus abdominal muscles in the plank might not be sensitive enough to be influenced by the minor changes in the spinal stability, antagonist muscles that were substantially less activated compared to agonist muscles might be more susceptible to the changes in stability from the static deep flexion.

If the changes in the spinal stability can be better detected or identified from the recruitment patterns of antagonist muscles, the evaluation of antagonist muscles may work better as an efficient and more reliable method for assessing the health status of the low back, specifically when monitoring minor changes in spinal stability in daily activities. The findings of this pioneering study, however, need further evaluation in future research with various test conditions. Specifically, it is of interest whether the results of this study would hold true for other modes of spine health damage such as the development of muscle fatigue.

## Conclusions

Results of this study suggest the possibility of using surface EMG in the evaluation of spinal stability and low back health status in simple exercises that can be conducted in daily life. The sensitivity of the surface EMG in identifying the changes in the spinal stability can be improved when the EMG of antagonist muscles are measured in plank or isometric back extension exercises. Decrements in the amplitude of antagonist muscles in the plank and isometric back extension may be indicative of decreased stability of the lumbar spine.
